# Assessing the impact of non-protein nitrogen or rumen undegradable protein supplementation on rumen bacterial diversity and ruminal fermentation in grazing steers during the dry season

**DOI:** 10.3389/fmicb.2025.1670636

**Published:** 2025-10-06

**Authors:** Ana Veronica Lino Dias, Yury Tatiana Granja-Salcedo, Juliana Duarte Messana, Karine Dalla Vecchia Camargo, Kênia Larissa Gomes Carvalho Alves, Elisabeth Victória Alves Machado, Milena Tavares Lima Constancio, Ricardo Andrade Reis, Telma Teresinha Berchielli

**Affiliations:** ^1^Department of Animal Sciences, School of Agricultural and Veterinary Sciences, São Paulo State University (UNESP), Jaboticabal, SP, Brazil; ^2^Corporación Colombiana de Investigación Agropecuaria, AGROSAVIA, EI Nus Research Center, San Roque, Antioquía, Colombia

**Keywords:** corn gluten, fiber digestibility, fibrolytic bacteria, rumen ammonia, tropical forage, urea

## Abstract

**Introduction:**

This study aimed to evaluate the effects of sources of non-protein nitrogen (NPN) or rumen undegradable protein (RUP) as supplements on intake, nutrient digestibility, fermentation parameters, and ruminal microbiota in Nellore steers grazing during the dry season.

**Methods:**

The experiment was conducted during the dry season from September to October 2018, in a grazing area of *Urochloa brizantha (A. Rich.) Stapf.* cv. *Xaraés.* Eight rumen-and duodenum-cannulated Nellore steers were used in a 4 × 4 Latin square design (2 treatments and 2 periods) balanced for residual effects. The treatments included (1) mineral salt with urea (SMU), formulated to meet 50% of the steer RDP requirement as NPN; and (2) supplementation with corn gluten meal (GLU; 0.3% of body weight) as a source of RUP, with added mineral salt.

**Results:**

GLU supplementation significantly increased supplement intake (*p* < 0.05) and tended to decrease the intake of forage NDF. Although GLU supplementation improved NDF digestibility, no significant differences were observed in the digestibility of DM, OM, or CP between the treatments (*p* > 0.05). GLU supplementation significantly increased the relative abundance of several genera, including *Ruminococcus 1*, *Ruminococcus 2*, *Erysipelotrichaceae UCG-004*, *Thermoplasmatales Incertae Sedis*, *Lachnospiraceae XPB1014*, *Anaeroplasma* spp., *Anaerotruncus* spp. and *Eubacterium ruminantium* (*p* < 0.05). The PCA biplot revealed positive associations between GLU supplementation and greater ruminal abundance of *Prevotellaceae UCG-004* and Bacteroidetes, as well as with higher concentrations of butyrate, propionate, and valerate acids.

**Discussion:**

Supplementation with GLU as a source of RUP in grazing steers during the dry season modulated the rumen microbiota by increasing the abundance of key fibrolytic bacteria and improved fiber digestibility.

## 1 Introduction

Forages in tropical climates present significant challenges for beef cattle production, especially during the dry season when forage quality declines, resulting in low crude protein (CP) content and higher fiber indigestible fractions (Franco et al., 2017). The breakdown of ingested fiber in ruminants is not endogenous but mediated by a complex ruminal microbiome, which drives dietary fermentation (Koike and Kobayashi, 2009). Diets containing less than 7% crude protein (CP) significantly impair ruminal microbial activity due to nitrogen (N) limitation, as N serves as an essential substrate for microbial protein synthesis (Poppi and McLennan, 1995; Sampaio et al., 2009). This limitation limits fiber degradation, thereby reducing forage intake owing to slower passage rates and digestibility (Silva et al., 2009). Supplementing ruminant diets with nitrogenous compounds while grazing low-quality pastures can promote fiber degradation by providing ruminal ammonia N (Brooks et al., 2012; Batista et al., 2016). Microorganisms require ammonia N to synthesize the protein components of their cell walls, whereas ruminants also need rumen undegradable protein (RUP) and microbial protein (Firkins et al., 2007). However, non-protein nitrogen (NPN) supplementation may lead to ruminal ammonia N accumulation beyond microbial utilization (Bach et al., 2005). Excess ammonia N is converted to urea and then excreted, resulting in nitrogen loss that incurs a substantial energetic cost. This is compounded by the rumen’s inherently low ATP yield, which further constrains microbial growth efficiency and fermentative activity (Oliveira et al., 2020).

The richness and diversity of the rumen microbiota are influenced by dietary composition and are linked to nutrient utilization in ruminants (McLoughlin et al., 2020; Alves et al., 2020). In ruminants with high forage intake, energy deficiency may occur, and N recycling may be maximized by rumen microbes, which can reduce the efficiency of microbial protein synthesis (Poppi and McLennan, 1995; Clark et al., 1992). Rumen microbiota diversity is also related to N retention efficiency; a higher relative abundance of genera such as *Anaerohabdus* spp., *Succinimonas* spp., *Endomicrobium* spp., and *Eubacterium* spp. has been correlated with steers with high N retention (Alves et al., 2020). Changes in the metabolic activity in the rumen may be associated with variations in the abundance of different bacterial groups. Steers fed Tifton 85 hay supplemented with casein as a source of rumen-degradable protein (RDP), presented lower richness and diversity of some species of the phylum Bacteroidetes because of the increase in microbial populations with fewer species involved in protein metabolism (Bento et al., 2016). The replacement of NPN by protein-N increased methane production *in vitro*, attributed to differences in the use of the amino acids and shifts in microbiota population (Vanegas et al., 2017).

However, our previous study showed that when steers grazed low-quality forages, NPN supplementation did not increase microbial protein synthesis, and RUP supplementation did not improve the total amino acid flow to the duodenum (Dias et al., 2023), highlighting the need for further investigation. Therefore, a comprehensive evaluation of the rumen microbiome is essential, as its composition influences animal metabolism (Firkins et al., 2007). Studies that clarify how RUP supplementation affects the rumen microbiome during the dry season are necessary to develop effective supplementation strategies for cattle under grazing conditions. Furthermore, the source of RUP as a supplement was expected to shift the rumen microbiota composition, improving fermentative activity compared to a mineral supplement containing NPN. This study aimed to evaluate the effects of sources of NPN and RUP as supplements on intake, nutrient digestibility, fermentation parameters, and ruminal microbiota in Nellore steers grazing during the dry season.

## 2 Materials and methods

### 2.1 Experimental area and animals

The experiment was conducted during the dry season from September to October 2018, in a grazing area of *Urochloa brizantha (A. Rich.) Stapf.* cv. *Xaraés* located at a latitude of 48°18′58″W, longitude 21°15′22” S, and an altitude of 595 m. According to the international Köppen classification, the climate is categorized as tropical type Aw characterized by summer rains and a relatively dry winter. The average weather recorded data was 64.9 mm of precipitation and an average temperature of 22.9 °C in September, and 157 mm of rainfall with an average temperature of 24.4 °C in October.

Eight rumen- and duodenum-cannulated Nellore steers (*Bos taurus indicus*), with an average body weight of 263 ± 48.69 kg (12 ± 2 months old), were used in a 4 × 4 Latin square design (2 treatments and 2 periods) balanced for residual effects. The animals were distributed across eight paddocks of 1.8 hectares each, with automatic waterers and covered feeders for supplement provision. The grazing method was continuous grazing with variable stocking rates according to the “put-and-take” technique (Mott and Lucas, 1952) to achieve the pasture height target of 27 cm.

### 2.2 Ethical approval

All procedures complied with the ethical principles for animal experimentation established by the Brazilian College of Animal Experimentation and were approved by the Ethics Committee on Animal Use (CEUA) of the Faculty of Agricultural and Veterinary Sciences – UNESP, Jaboticabal/SP (protocol no. 16.688/16). All animals were monitored throughout the experimental period, and no negative effects on health, behavior, or feed intake were observed due to the supplementation strategies.

### 2.3 Feeding and treatment protocol

The treatments included (1) mineral salt with urea (SMU), formulated to meet 50% of the steer RDP requirement as NNP; and (2) supplementation with corn gluten meal (GLU; 0.3% of body weight) as a source of RUP, with added mineral salt (Table 1). Supplements were provided daily at 09:00 h, and weekly adjustments were made to the amount offered. GLU was selected as RUP source due to its high crude protein content, substantial RUP fraction (Table 1), and its content of essential amino acids valine and leucine (Dias et al., 2023).

**Table 1:** Dry matter and chemical composition of *Urochloa brizantha (A. Rich.) Stapf.* cv. *Xaraés* forage and experimental supplements.

Item	Forage*		Supplements	
	SMU	GLU	SMU	GLU
DM, % as-fed	74.68	76.51	59.00	91.21
Ash, % DM	5.91	5.93	0.33	2.62
OM, % DM	94.10	94.07	99.67	97.38
CP, % DM	5.41	5.61	222.75	58.75
RDP, % CP	63.41	63.19	100	30.30
RUP, % CP	36.59	36.81	-	69.70
EE, % DM	1.37	1.21	-	1.71
NFC, %DM	17.03	16.66	-	28.94
NDFap, % DM	65.12	65.83	-	-
NDFi, % DM	29.77	30.75	-	1.71
ADF, % DM	36.63	37.49	-	2.22

* Dry season from September to October 2018. SMU = Mineral salt with urea as source of non-protein nitrogen; GLU, corn gluten meal as rumen undegradable protein source plus mineral salt; DM, dry matter; OM, organic matter; CP, crude protein; RDP, rumen degradable protein; RUP, rumen undegradable protein estimated based on the protein fractions (Licitra et al., 1996); EE, ether extract; NFC, non-fiber carbohydrates; NDFap, Neutral detergent fiber expressed exclusive of residual ash and protein; iNDF, indigestible neutral detergent fiber; ADF, acid detergent fiber.

The supplements were designed to promote an average daily gain (ADG) of 0.350 kg. Nutritional requirements (kg/day) were dry matter (DM) = 4.58 kg, total digestible nutrients (TDN) = 2.82 kg DM, crude protein (CP) = 0.56 kg DM, and RDP = 67.86% of CP (Valadares Filho et al., 2016).

### 2.4 Forage characterization and nutritional analysis

Average canopy height was evaluated weekly by measuring 80 random points per hectare using a graduated ruler (Barthram, 1985). This measurement was used to adjust the stocking rates and ensure that the pasture height remained at 27 cm. Five representative points with average heights were selected from each paddock. At each point, all forage within a 0.25 m^2^ frame was cut to a height of 5 cm. The collected samples were weighed and separated into leaf and stem + sheaths to determine their morphological fractions. The samples were then oven-dried under forced air circulation at 55 °C for 72 h. The final dry weight was multiplied by the paddock area to estimate total forage mass.

The pasture was sampled every 28-d using a simulated grazing technique (Halls, 1954) to evaluate the chemical composition of the forage. The samples of each supplement were collected every 3 days. The feed samples were analyzed for dry matter (DM; method 934.01), organic matter (OM; method 942.05), and ether extract (EE; method 954.02) according to AOAC (1995) guidelines. Neutral detergent fiber (NDF) content was determined according to Mertens (2002), adapted for the Ankom200 Fiber Analyzer, and later corrected for ash and protein. The CP content was determined using a combustion N analyzer (Leco FP-528 Carbon/N Analyzer, Leco Instruments Inc., St. Joseph, MI); method 990.13 (AOAC, 2005).

### 2.5 Feed intake and nutrient digestibility

Nutrient intake and digestibility were estimated based on fecal output data using chromium oxide (Cr₂O₃) and indigestible neutral detergent fiber (iNDF) as external and internal markers, respectively. To estimate fecal output, Cr₂O₃ packed in paper cartridges was administered directly into the rumen at a dosage of 8 g per animal per day during the last 12 days of each experimental period (7 days for marker stabilization and 5 days for fecal collection). Fecal samples were collected from each steer over five consecutive days at varying times (day 1:9:00 a.m.; day 2:1:00 p.m.; day 3:5:00 p.m.; day 4:9:00 p.m.; and day 5:6:00 a.m.), on days 24, 25, 26, 27, and 28 of each experimental period. After collection, the samples were dried in an oven at 55 °C for 72 h, homogenized, and ground in a Willey-type mill (Thomas Scientific, Swedesboro, NJ, USA) to 1 mm for Cr analysis and 2 mm for iNDF determination.

Fecal chromium concentration was determined using atomic absorption spectrophotometry (Williams et al., 1962). Fecal output was estimated by fractioning the amount of Cr₂O₃ supplied to the rumen and its concentration in feces. Fecal output = Cr₂O₃ supplied*/*[Cr₂O₃ infeces */*DM105 ∘C] where FP = fecal production obtained by Cr₂O₃ g (DM/day); Cr₂O₃ supplied = amount of Cr₂O₃ supplied to the animals per day; Cr₂O₃ in feces = percentage of Cr₂O₃.

Nutrient digestibility was estimated using the iNDF content of the forage samples collected via simulated grazing, supplements, and feces. The iNDF was determined after *in situ* rumen incubation for 288 h (Valente et al., 2011). The samples were then subjected to neutral detergent extraction using an Ankom Fiber Analyzer (Ankom Inc., Fairport, NY, USA) (Mertens, 2002). The total dry matter intake was calculated as the sum of forage and supplement intake. Nutrient digestibility (ND) was calculated as follows: digestibility (%) = [(intake-fecal excretion) / intake] × 100.

To estimate dry matter flow and calculate the rumen-degraded organic matter (RDOM), duodenal digesta was collected via duodenal cannula four times per day (day 1: 2:00, 8:00, 14:00, and 20:00; day 2: 5:00, 11:00, 17:00, and 23:00), homogenized by animal and period, then dried at 55 °C for 72 h for chemical and iNDF analyses. Duodenal dry matter flow was determined using iNDF as an indicator (Udén et al., 1980).

### 2.6 Rumen fermentation and metabolites

On day 28 of each experimental period, rumen fluid samples were manually collected through the rumen cannula at 0 h (before supplementation) and 6, 12, 18, and 24 h after supplementation. Approximately 50 mL of rumen fluid was filtered through two layers of cotton cloth and immediately used for pH determination using a digital potentiometer (Nova Tecnica, PHM, Piracicaba, SP, Brazil). Then, 40 mL was stored at −20 °C for ammonia N (N-NH₃) analysis by micro-Kjeldahl distillation (Fenner, 1965). A 20 mL aliquot was centrifuged at 13,000 × g for 30 min at 4 °C, and the supernatant was separated, isolated, and stored at −15 °C for later determination of short-chain fatty acids (SCFA) concentration by gas chromatography. SCFA concentration was determined using a gas chromatograph (GC2014, Shimadzu Corporation, Kyoto, Japan) equipped with a capillary column (HP INNOWax; 30 m, 0.32 mm, 0,50 μm, Agilent Technologies, Colorado, USA).

Spot urine samples were collected daily on days 25–28 at 02:00, 11:00, 15:00, and 21:00 (Silva et al., 2018). Samples were filtered through gauze; a 50 mL aliquot was frozen directly, and 10 mL was diluted in 40 mL of 0.036 N sulfuric acid to prevent N loss and stored at −20 °C until further analysis.

Creatinine concentration in urine was determined by an enzymatic method from the alkaline picrate reaction using a commercial kit (K016–1, Bioclin, Belo Horizonte, MG, Brazil). Daily urinary creatinine excretion (CE) was estimated using the equation proposed by Silva et al. (2012) for Nellore steers:

$$\mathrm{CE}\;(g/d)=0.0345\times {\left(\mathrm{BW}\right)}^{0.9491}$$

Where CE is creatinine excretion and BW is body weight (kg).

The urinary excretion of purine derivatives (PDE, mmol/d) was calculated as the sum of uric acid and allantoin excreted in the urine. Allantoin as described by Chen and Gomes (1992), and uric acid using a commercial kit (Ácido Úrico Liquiform®, Cat. no. 140), following the manufacturer’s instructions. Purine derivatives absorption (PDA, mmol/d) was calculated using the equation proposed by Verbic et al. (1990) for steers,

$$\mathrm{PDA}=0.85(\mathrm{PDE})+0.385\times {\mathrm{BW}}^{0.75}$$

Where 0.85 is the recovery rate of absorbed purines and 0.385 × BW^0.75^ represents the endogenous purine excretion per unit of metabolic body size.

Rumen microbial protein synthesis (MPS) was estimated using PDA as described by Chen and Gomes (1992).

MicrobialN(mg/day)=(70×PDA)/(0.83×0.134)

where 70 is the N content in purines (mg N/mmol), 0.134 corresponds to the purine N to total N ratio in bacteria (Valadares et al., 1999), and 0.83 is the digestibility of microbial purines. The MPS was obtained by multiplying Microbial N by 6.25. Microbial efficiency (EMPS) was expressed as grams of MPS per kilogram of RDOM (Zinn and Owens, 1986).

### 2.7 Rumen bacterial analysis

#### 2.7.1 Rumen content sampling

On the 28th day of each experimental period, rumen samples for bacterial diversity analysis were collected via rumen cannula, including both solid and liquid digesta fractions, before supplementation. Approximately 50 g of content from the ventral sac of the rumen was collected and stored in DNA- and RNA-free Falcon tubes, then immediately transported to the laboratory in ice-filled coolers. In the laboratory, 50 mL of PBS solution (pH 7.4, at 4 °C) was added to the samples, which were then processed to obtain a microbial pellet (Granja-Salcedo et al., 2017).

#### 2.7.2 DNA extraction and sequencing

DNA extraction was performed using 200 mg of the pellet and the Quick-DNA™ Fecal/Soil Microbe Miniprep Kit (Zymo Research), following the manufacturer’s instructions and using the FastPrep^®^ system for cell lysis. DNA yield was assessed via spectrophotometry (NanoDrop^®^ ND-1000) and fluorometry (Qubit^®^ 3.0, using the Qubit^®^ dsDNA Broad Range Assay Kit). DNA purity was evaluated using absorbance ratios (A_260/280_ and A_260/230_), and DNA integrity was confirmed by electrophoresis in 0.8% agarose gel stained with SYBR™ Gold.

The V4/V5 region of the bacterial 16S rRNA gene was amplified via PCR using forward primer 515F (5’-GTGNCAGCMGCCGCGGTAA-3′) and reverse primer 926R (5’-CCGYCAATTYMTTTRAGTTT-3′) described by Caporaso et al. (2011). The PCR mix contained 20 ng of DNA template, 1.25 mM MgCl₂, 200 μM dNTPs, 1.0 U Taq DNA polymerase, 1 × PCR buffer, 10 pmol of each primer, and ultrapure water to a final volume of 20 μL. PCR was run in duplicate under the following conditions: 95 °C for 3 min; 30 cycles of 95 °C for 30 s, 53.8 °C for 30 s, 72 °C for 45 s; and a final extension at 72 °C for 10 min. PCR products were verified on a 1% agarose gel, and fragment size was compared to a 1 kb Plus DNA Ladder. Amplicons were purified using the Zymoclean™ Gel DNA Recovery Kit, pooled in equimolar amounts, and sequenced using the Illumina MiSeq platform (MiSeq Reagent v2 kit, 2 × 250 bp, Illumina Inc., NY, USA).

#### 2.7.3 Analysis of sequencing data

Sequence data were processed using the QIIME software package (Bolyen et al., 2019). Reads longer than 250 bp with an average Phred quality score >25 were selected using the q2-demux plugin and denoised with DADA2 (Callahan et al., 2016). Taxonomy was assigned to amplicon sequence variants (ASVs) using the QIIME 2 feature classifier (Bokulich et al., 2018), and OTUs (Operational Taxonomic Units) were grouped at 97% similarity. Rarefaction curves, OTU counts, and taxonomic assignments were analyzed at different taxonomic levels using the SILVA 128 database. Richness estimators (ACE and Chao1) and alpha/beta diversity indices were calculated (Faith, 1992; Lozupone and Knight, 2005). Predicted functional profiles of microbial communities were generated using PICRUSt workflow (Langille et al., 2013). Predicted functional genes were categorized into KEGG Orthology (KO) pathways.

### 2.8 Statistical analysis

Statistical analyses were performed using R software (version 3.6.3). Data normality and homogeneity of variance were assessed using Shapiro–Wilk and Bartlett’s tests, respectively.

Intake and digestibility data were analyzed using a duplicated 2 × 2 Latin square design balanced for residual effects. The model included treatment as a fixed effect, Latin square, animal, period, and residual error as random effects.

Rumen parameters were also analyzed using a duplicated 2 × 2 Latin square design balanced for residual effects, with repeated measures over time (sampling time). The model included fixed effects of treatment, sampling time, and their interaction (treatment × time), and random effects of Latin square, animal, period, and residual error. Post-hoc comparisons were performed using Tukey’s HSD test when ANOVA revealed significant effects (*p* ≤ 0.05).

Rumen bacterial and archaeal richness, diversity indices, relative abundance, and KEGG functional predicted pathways were compared between NPN and RUP treatments using the paired Wilcoxon test. Log₂ fold change (log₂ FC) was used to quantify the magnitude and direction of differences in microbial abundances between treatment groups. Principal Component Analysis (PCA) was used to extract key microbial OTUs and KEGG functional predicted pathways in animals supplemented with NPN or RUP using the FactoMineR package.

*p*-values ≤ 0.05 were considered significant, and values between 0.05 and 0.10 were considered trends in all analyses.

## 3 Results

### 3.1 Feed intake and nutrient digestibility

No differences were observed between SMU and GLU for forage mass, leaf mass, or leaf-to-stem ratio (Table 2; *p* > 0.05). Forage height showed a tendency toward significance (*p* = 0.052), with SMU generating a taller canopy compared to GLU.

**Table 2:** Mean values of quantitative characteristics and forage height of *Urochloa brizantha (A. Rich.) Stapf. cv. Xaraés* paddocks during the experimental period*.

Supplement	Forage mass (kg DM/ha)	Leaf mass (kg DM/ha)	Canopy Height (cm)	Leaf (% DM)	Stem (% DM)
SMU	5983.56	2848.28	30.19	50.55	49.45
GLU	5302.91	2745.04	28.08	53.51	46.49
SEM	416.92	160.95	1.39	3.76	3.76
*P*-value	0.153	0.787	0.052	0.345	0.349

* Dry season from September to October 2018. SMU, Mineral salt with urea as source of non-protein nitrogen; GLU, corn gluten meal as rumen undegradable protein source plus mineral salt; SEM, Standard error of the mean; DM, dry matter.

Treatments (SMU vs. GLU) did not affect total dry matter intake or organic matter intake (Table 3; *p* > 0.05). However, GLU supplementation increased supplement intake (0.34 vs. 0.11 kg/d; *p* = 0.013) while tending to decrease forage NDF intake (2.09 vs. 2.01 kg/d; *p* = 0.069). Ether extract intake was higher for GLU (48.7 vs. 36.3 g/d; *p* = 0.043). In addition, GLU supplementation improved NDF digestibility (53.36 vs. 43.61%; *p* = 0.017), while no differences were observed for DM, OM, or CP digestibility between treatments (*p* > 0.05).

**Table 3:** Intake and apparent digestibility of nutrients in Nellore steers grazing *Urochloa brizantha (A. Rich.) Stapf. cv. Xaraés* during the dry season and supplemented with SMU or GLU.

Intake	Supplement		SEM	p-value
	SMU	GLU		
Total DM, % BW	1.05	1.00	0.070	0.804
Total DM, kg/d	2.75	2.61	0.180	0.910
Supplement DM, kg/d	0.11	0.34	0.061	0.013
Forage DM, kg/d	2.65	2.26	0.157	0.244
OM, kg/d	2.51	2.46	0.151	0.631
CP, kg/d	0.43	0.35	0.055	0.516
NDF, kg/d	2.01	2.09	0.027	0.069
EE, g/d	36.3	48.7	3.400	0.043
Total Digestibility, % DM				
DM	28.56	30.32	4.742	0.505
OM	35.13	39.06	4.679	0.509
CP	47.71	40.82	8.634	0.713
NDF	43.61	53.36	2.135	0.017

SMU, Mineral salt with urea as source of non-protein nitrogen; GLU, corn gluten meal as rumen undegradable protein source plus mineral salt; SEM, Standard error of the mean; DM, dry matter; BW, body weight; OM, organic matter; CP, crude protein; NDF, Neutral detergent fiber; EE, ether extract.

### 3.2 Rumen fermentation and metabolites

The sampling time × supplement interaction did not affect the rumen fermentation parameters (Table 4; p > 0.05). Treatments (SMU vs. GLU) had no effect on rumen pH, ammonia-N, total SCFA concentration, MPS and EMPS (*p* > 0.05). However, GLU supplementation increased the molar proportions of isobutyrate (1.31 vs. 1.48%), isovalerate (1.56 vs. 1.73%), and valerate (0.83 vs. 0.88%) in the rumen (*p* < 0.05).

**Table 4:** Rumen fermentation parameters in Nellore steers grazing *Urochloa brizantha (A. Rich.) Stapf. cv. Xaraés* during the dry season and supplemented with SMU or GLU.

Item	Supplement		EPM	P-value		
	SMU	GLU		Time	Supplement	T × S
pH	6.89	6.93	0.214	<0.001	0.442	0.464
N-NH₃, mg/dL	24.41	23.09	0.331	<0.001	0.326	0.624
Total SCFA, mmol/L	95.91	93.02	3.413	<0.001	0.220	0.964
Acetate, % SCFA	68.21	67.89	1.922	<0.001	0.323	0.555
Propionate, % SCFA	17.60	17.43	0.510	<0.001	0.488	0.721
*iso*Butyrate, % SCFA	1.31	1.48	0.049	<0.001	<0.001	0.143
Butyrate, % SCFA	10.46	10.46	0.315	0.036	0.640	0.477
*iso*Valerate, % SCFA	1.56	1.73	0.063	<0.001	<0.001	0.227
Valerate, % SCFA	0.83	0.88	0.028	<0.001	0.041	0.245
Acetate-Propionate ratio	3.90	3.95	0.127	<0.001	0.481	0.679
MPS, g/d	112.36	83.32	13.754	-	0.678	-
EMPS, g of MPS/kg RDOM	161.37	115.89	52.643	-	0.150	-

SMU, Mineral salt with urea as source of non-protein nitrogen; GLU, corn gluten meal as rumen undegradable protein source plus mineral salt; SEM, Standard error of the mean; SCFA, short-chain fatty acids; N-NH₃, ammonia nitrogen; MPS, rumen microbial protein synthesis; EMPS, microbial efficiency; RDOM, rumen-degraded organic matter. T × S, supplement and sampling time interaction.

The sampling time influenced all ruminal fermentation parameters (*p* < 0.05; Table 4). At 6 and 12 h after supplementation, the pH and acetate proportion were lower (*p* < 0.001), whereas the total SCFA and proportions were higher than at other sampling time (*p* < 0.001). The ammonia-N concentration was highest at 6 h and lowest at 18 h (*p* < 0.001). The butyrate proportion was lowest at 24 h compared to that at 6 and 12 h (*p* < 0.001).

### 3.3 Rumen bacterial analysis

Sequencing and processing of the samples yielded an average of 32,298 sequences per sample. Microbial community analysis revealed no differences between SMU and GLU supplementation groups in either alpha diversity metrics (ACE = SMU 98.99 ± 11.30 vs. GLU 98.18 ± 12.83; Chao1 = SMU 96.00 ± 11.00 vs. GLU 96.00 ± 13.00; *p* > 0.79), diversity estimators (Shannon-Weiner = SMU 3.57 ± 0.15 vs. GLU 5.54 ± 0.11; Simpson = SMU 9.51 ± 0.12 vs. GLU 9.45 ± 0.04; Fisher = SMU 12.52 ± 1.11 vs. GLU 12.96 ± 1.29; *p* > 0.68), or microbial domain composition of Archaea (SMU 3.18 ± 1.90 vs. GLU 3.02 ± 1.73; *p* = 0.81) and Bacteria (SMU 81.74 ± 3.11 vs. GLU 81.96 ± 1.69; *p* = 0.57).

A total of 17 bacterial phyla and one archaeal phylum were identified (Table 5), along with 38 classes, 64 orders, 105 families, 237 genera, and 407 species. At the phylum level, GLU supplementation altered bacterial populations by reducing the relative abundance of Firmicutes (*p* = 0.018), increasing Chloroflexi (*p* = 0.046), and showing a trend toward enrichment of Synergistetes (*p* = 0.096).

**Table 5:** Rumen bacterial and archaeal phylum abundance (median ± interquartile range of abundance) in Nellore steers grazing *Urochloa brizantha (A. Rich.) Stapf. cv. Xaraés* during the dry season and supplemented with SMU or GLU.

Domain	Phylum	Supplement		*P*-value
		SMU	GLU	
Archaea	Euryarchaeota	3.09 ± 1.72	3.18 ± 1.90	0.812
Bacteria*	Actinobacteria	0.68 ± 0.61	0.64 ± 0.28	0.937
	Bacteroidetes	29.53 ± 4.30	29.98 ± 2.35	0.468
	Chloroflexi	0.89 ± 0.44	1.32 ± 0.27	0.046
	Cyanobacteria	0.04 ± 0.09	0.01 ± 0.06	0.675
	Elusimicrobia	0.01 ± 0.01	0.01 ± 0.04	0.583
	Fibrobacteres	0.30 ± 0.12	0.31 ± 0.45	0.968
	Firmicutes	48.17 ± 3.37	44.92 ± 2.84	0.018
	Lentisphaerae	0.05 ± 0.02	0.01 ± 0.03	0.294
	Planctomycetes	0.13 ± 0.06	0.11 ± 0.06	0.833
	Proteobacteria	1.29 ± 0.24	1.20 ± 0.54	0.937
	SR1 Absconditabacteria	0.11 ± 0.55	0.05 ± 0.08	0.375
	Saccharibacteria	0.01 ± 0.01	0.01 ± 0.04	0.418
	Spirochaetae	1.25 ± 0.44	1.29 ± 0.84	0.812
	Synergistetes	0.01 ± 0.17	0.26 ± 0.24	0.096
	Tenericutes	0.48 ± 0.54	0.58 ± 0.31	0.937
	Verrucomicrobia	0.43 ± 0.12	0.68 ± 0.62	0.375

* Fusobacteria were identified in one sample of SMU (abundance of 0.034). SMU, Mineral salt with urea as source of non-protein nitrogen; GLU, corn gluten meal as rumen undegradable protein source plus mineral salt.

**Figure 1:**
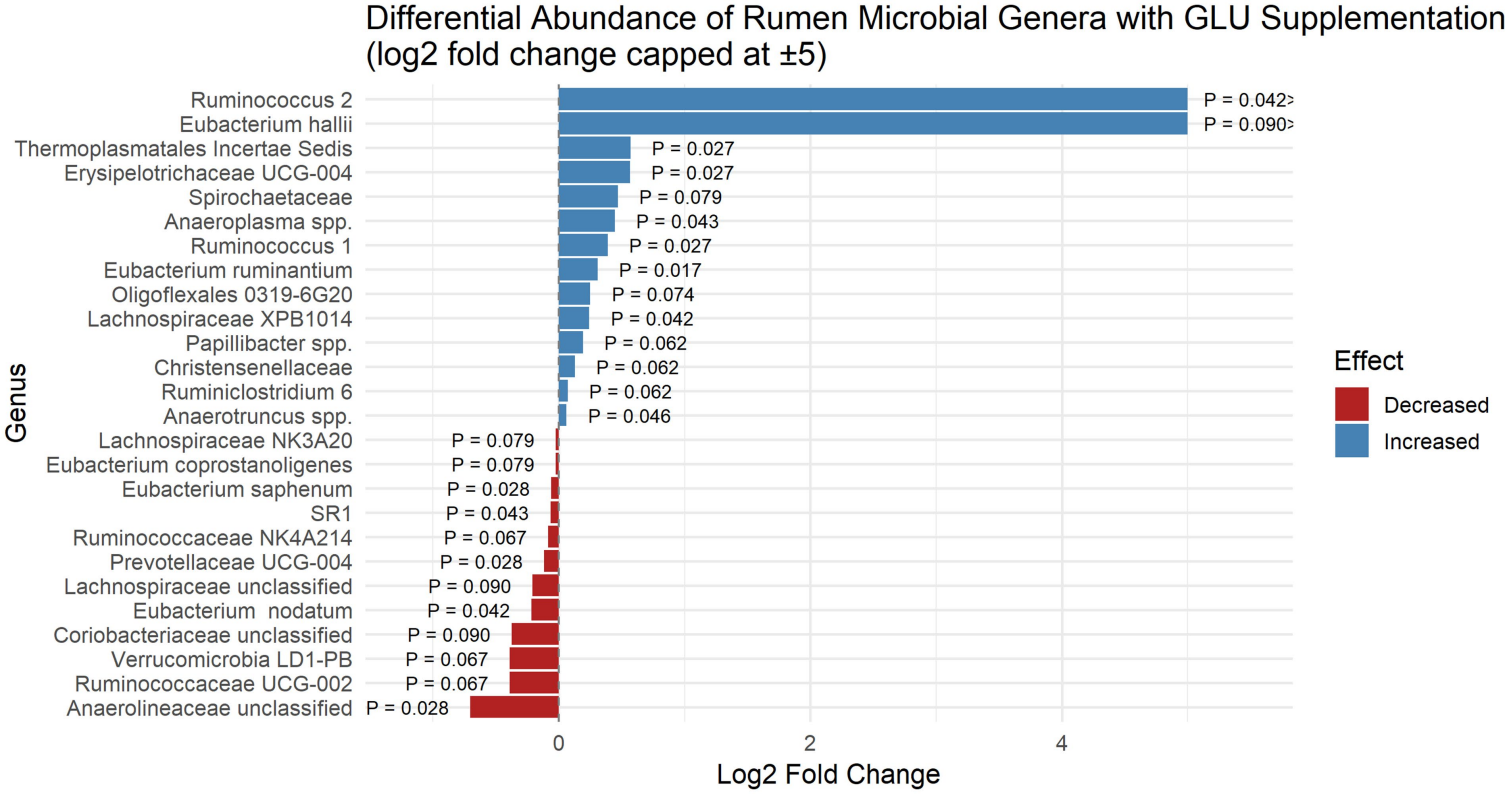
Differential abundance (log₂ fold change) of rumen microbial genera in grazing Nellore steers supplemented with corn gluten meal as rumen undegradable protein source plus mineral salt (GLU) during the dry season. Bars represent genera that were affected by the treatment (*p* < 0.10). Positive and negative values indicate increased or decreased relative abundance, respectively, in the GLU group compared to the non-protein nitrogen (SMU). *p*-values are shown above each bar. The log₂ fold change for *Ruminococcus 2 and Eubacterium hallii* were approximately 30.6 and 12.4 and were visually capped at 5 for improved readability of the plot.

At the genus level, GLU supplementation significantly altered the rumen microbial composition by reducing the abundance of several bacterial taxa (Figure 1), including *Eubacterium saphenum* (*p* = 0.028), *Eubacterium nodatum p* = 0.042, *Prevotellaceae* UCG-004 (*p* = 0.028, FC = -0.117), *Anaerolineaceae* unclassified (*p* = 0.028, FC = −0.703), SR1 (*Absconditabacteria*) (*p* = 0.043, FC = −0.066), *Ruminococcaceae* NK4A214 (*p* = 0.067), *Ruminococcaceae* UCG-002 (p = 0.067), *Verrucomicrobia* LD1-PB (*p* = 0.067), *Lachnospiraceae* NK3A20 (*p* = 0.079) and *Lachnospiraceae* unclassified members (*p* = 0.090), *Coriobacteriaceae* unclassified members (*p* = 0.090).

Conversely, GLU supplementation increased the relative abundance of several genera (Figure 1), including *Ruminococcus 1* (*p* = 0.027), *Ruminococcus 2* (*p* = 0.042), *Erysipelotrichaceae* UCG-004 (*p* = 0.027), *Thermoplasmatales* Incertae Sedis (*p* = 0.027), *Lachnospiraceae* XPB1014 (*p* = 0.042), *Anaeroplasma* spp. (*p* = 0.043), *Ruminiclostridium 6* (*p* = 0.062), *Christensenellaceae* (*p* = 0.062), *Papillibacter* spp. (*p* = 0.062), *Oligoflexales* 0319-6G20 (*p* = 0.074), *Spirochaetaceae* (*p* = 0.079), *Eubacterium hallii* (*p* = 0.090), *Anaerotruncus* spp. (*p* = 0.046) and *Eubacterium ruminantium* (*p* = 0.017).

**Figure 2:**
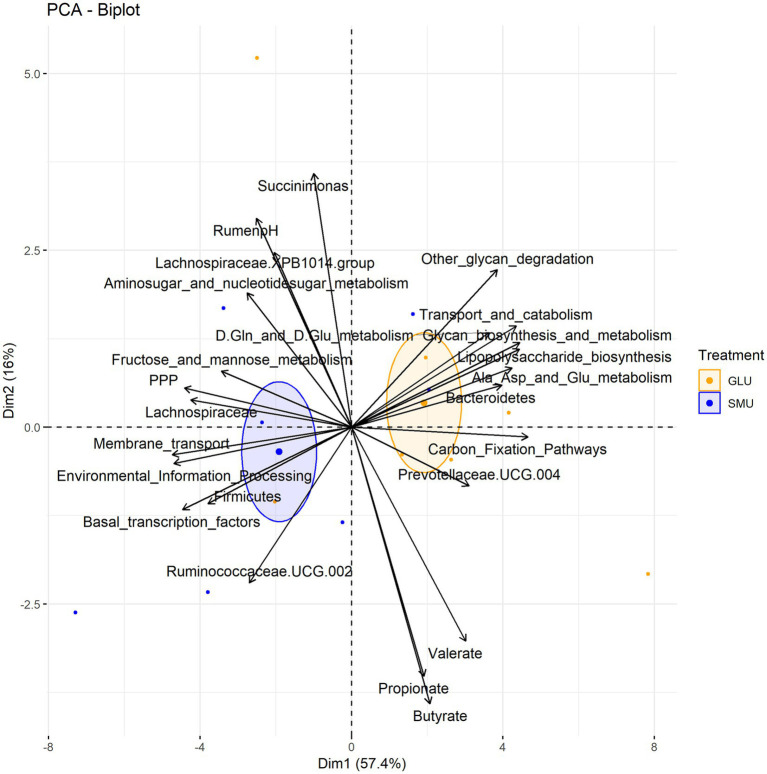
Principal component analysis (PCA) biplot showing the correlation of microbial taxa, KEGG metabolic predicted pathways, and fermentation parameters in grazing Nellore steers supplemented with corn gluten meal as rumen undegradable protein source plus mineral salt (GLU) or mineral salt with urea as source of non-protein nitrogen (SMU) during the dry season. Vectors represent the contribution and direction of each variable, while points represent individual animals colored by treatment group: GLU (orange) and SMU (blue). PPP, Pentose Phosphate Pathway.

The first two principal components (Dim.1 and Dim.2) of PCA account for 76.4% of the variability in bacterial communities, ruminal fermentation parameters, and predicted functional profiles across treatments (Figure 2). The PCA biplot revealed a clear separation between the GLU and SMU treatments. The GLU clustered toward the right side of the plot, associated with positive values on Dim.1 such as increased abundances of *Prevotellaceae UCG-004* (r = 0.64) and Bacteroidetes phylum (r = 0.82), as well as with higher concentrations of butyrate (r = 0.43), propionate (r = 0.40), and valerate (r = 0.64) acids. These shifts were accompanied by positive correlations with predicted pathways related to glycan biosynthesis and metabolism (r = 0.92), lipopolysaccharide biosynthesis (r = 0.92), carbon fixation (r = 0.96), amino acid metabolism (e.g., *Ala, Asp, and Glu metabolism*, r = 0.88), Other glycan degradation (r = 0.79).

In contrast, the SMU group, clustering negatively along Dim.1, was associated with increased abundance of Firmicutes phylum (r = −0.78), *Ruminococcaceae UCG-002* (r = −0.56), and *Lachnospiraceae* (r = −0.88), as well as the predicted pathways membrane transport (r = −0.98), environmental information processing (r = −0.97), basal transcription factors (r = −0.92), pentose phosphate pathway (r = −0.91), Fructose and mannose metabolism (r = −0.71), and Aminosugar and nucleotidesugar metabolism (r = −0.56).

Finally, Dim.2 revealed positive correlations between Succinimonas (r = 0.74), Lachnospiraceae XPB1014 group (r = 0.51), and rumen pH (r = 0.61), while negative correlations were observed for SCFA including butyrate (r = −0.81), propionate (r = −0.73), and valerate (r = −0.62).

## 4 Discussion

The findings of this study partially support the initial hypothesis that supplementation with a RUP source would shift the rumen microbiota composition and improve rumen fermentation activity compared to a mineral supplement containing NPN. GLU supplementation, which provided RUP, significantly altered the rumen microbial community by increasing the relative abundance of key fibrolytic genera, higher concentrations of isobutyrate, isovalerate, and valerate, as an indicative of enhanced amino acid fermentation. Furthermore, PCA revealed that GLU supplementation was associated with increased activity in microbial predicted pathways related to glycan and lipopolysaccharide biosynthesis, carbon fixation, and amino acid metabolism, particularly involving alanine, aspartate, and glutamate. Although these microbial and metabolic responses point to a more active fermentative environment in GLU-supplemented steers, they did not translate into significant improvements in overall nutrient intake or total SCFA concentrations when compared to the SMU. This was likely due to the high indigestible fiber content of the forage, which limited energy availability and prevented further enhancement of MPS and its EMPS. Therefore, while the RUP-based GLU supplement did induce shifts in microbiota composition and metabolic potential, the expected improvements in fermentation and nitrogen utilization were constrained by forage quality, partially confirming this study hypothesis.

The quantitative characteristics of the forage were similar across treatments. As expected, the CP content during the experimental period was below 7%, which is considered the minimum threshold for optimal ruminal microbial activity (Ørskov, 2000). According to Detmann et al. (2009) protein supplementation in cattle grazing low-quality forages may enhance dry matter intake and improve energy balance by stimulating the growth of fibrolytic bacteria, which utilize ruminal ammonia as a N source for MPS during the degradation of forage carbohydrates (Firkins et al., 2007). In the present study, GLU supplementation significantly increased the relative abundance of *Ruminococcus 1*, *Ruminococcus 2, Ruminiclostridium 6,* and *Eubacterium ruminantium*, major rumen bacteria recognized as key fibrolytic bacteria involved in the breakdown of structural carbohydrates (Koike and Kobayashi, 2009). However, despite the provision of rumen RUP by GLU supplementation, no significant differences were observed in dry matter intake and digestibility when compared to SMU supplementation, except for EE intake and NDF digestibility, both of which were higher in animals supplemented with GLU. Similarly, (Corte et al., 2018) found no effects of replacing soybean meal with urea on the intake and total apparent digestibility in Nellore cattle. Nonetheless, the increased abundance of fibrolytic bacteria in the GLU group likely contributed to improved fiber digestibility, indicating a favorable microbial response that enhanced ruminal degradation of fibrous components. The higher EE intake observed in steers supplemented with GLU was expected, considering both the bromatological composition, with the GLU supplement containing 1.71% more EE than the SMU supplement, and the greater supplement intake observed in the GLU group (0.34 vs. 0.11 kg/d).

Despite the microbial shifts previously mentioned, no significant differences were observed between treatments in ruminal pH, ammonia-N concentration, total SCFA concentrations, MPS, or EMPS. These results suggest that the positive changes in microbial composition were not sufficient to enhance overall fermentation capacity or microbial protein yield under the conditions of this study. This may be due to the high proportion of indigestible fiber in the forage (NDFi 29.77–30.75% of DM), which limited energy availability and microbial proliferation, even in the presence of adequate nitrogen sources. The forage collected during the experimental periods was dry and fibrous, with a low nutritional value. Akin et al. (1987) reported that tropical grasses tend to have higher lignin content in parenchymal cell walls, which reduces digestibility, an effect also observed in the present study. In addition, Lempp (2013) showed that *Urochloa brizantha* cv. Xaraés has more lignified structures than other *Brachiaria* cultivars, which limits microbial colonization and degrades overall forage quality (Brito and Rodella, 2001). Furthermore, reduced forage degradability, which limits the release of fermentable sugars for microbial growth (Detmann et al., 2009), and increased rumen fill may have restricted intake (Allen, 1996) and fermentation. Thus, the high proportion of indigestible cell wall material of the forage limiting improvements in microbial efficiency and utilization of N (8; 44).

GLU contains nearly 3.2, 10.9 and 6.2% of valine, leucine, and proline, respectively (Dias et al., 2023). Steers supplemented with GLU showed higher ruminal concentrations of isobutyrate, isovalerate, and valerate, probably resulting from the deamination of these amino acids present in the GLU supplement. Similar findings were reported by Camargo et al. (2022) in grazing Nellore cattle supplemented with GLU during the rainy season. These branched-chain volatile fatty acids (BCVFA) are commonly produced through microbial amino acid catabolism and serve as growth factors for several fibrolytic bacteria (Firkins et al., 2024), supporting the observed enrichment of *Ruminococcus 1*, *Ruminococcus 2, Ruminiclostridium 6,* and *Eubacterium ruminantium* populations in GLU supplemented steers. Although GLU supplementation increased the relative abundance of fibrolytic bacteria and BCVFA concentrations, the energy derived from diet fibrous components was insufficient to support changes in SCFA production. In addition, the PCA results, indicating that GLU supplementation promotes a microbial and metabolic profile associated with enhanced fermentative activity and nutrient utilization, characterized by increased activity in predicted pathways such as glycan biosynthesis and metabolism, lipopolysaccharide biosynthesis, carbon fixation, and amino acid metabolism (e.g., alanine, aspartate, and glutamate metabolism), as well as the abundance of Bacteroidetes phylum and its genera *Prevotellaceae UCG-004*. The enrichment of predicted pathways related to alanine, aspartate, and glutamate metabolism in GLU supplemented steers suggests a greater reliance on microbial amino acid metabolism to meet ATP demands for maintenance and growth (Hackmann and Firkins, 2015). The amino acids provided by GLU may have supported the enrichment of Synergistetes phylum and other proteolytic or synergistic bacteria. Members of this phylum are strict anaerobes known for their involvement in amino acid, peptides and protein degradation (Jumas-Bilak and Marchandin, 2014).

PCA also revealed an upregulation of predicted pathways related to glycan biosynthesis and lipopolysaccharide production in GLU steers linked to the abundance of Bacteroidetes and *Prevotellaceae UCG-004*. Bacteria synthesize a wide array of glycans that are integral components of lipopolysaccharides, capsular polysaccharides, and antibiotic glycosides (Merritt et al., 2013). However, these associations are based on predicted functions rather than direct metagenomic evidence and should not be interpreted as causal. Members of the phylum Bacteroidetes, particularly those inhabiting the gut, are among the major producers of surface glycans (Coyne and Comstock, 2008). In addition, *Prevotellaceae UCG-004* has been associated with fiber degradation (Emerson and Weimer, 2017) and the production of acetate and butyrate (Jiang et al., 2022). While these microbial and functional responses suggest a more active fermentative environment, they were not sufficient to translate into significant improvements in overall nutrient digestibility, possibly due to limitations imposed by forage quality and fiber indigestibility (Nocek and Russell, 1988).

Leng (1990) reported that a minimum N-NH₃ concentration of 10 mg/dL is required to support optimal microbial growth. Detmann et al. (2010) further suggested that values around 15 mg/dL are ideal for maximizing the synthesis of microbial nitrogenous compounds in the rumen. In the present study, both supplements appeared to supply sufficient ammonia to meet microbial nitrogen requirements. This may have occurred not only through direct N supplementation but also via endogenous N recycling, particularly from the high RUP fraction of GLU reaching the intestine, which could be converted to urea and returned to the rumen via salivary or ruminal absorption pathways (Brake et al., 2010). This process is facilitated by ureolytic bacteria, adherent to the rumen epithelium, which hydrolyzes urea into ammonia N (Cheng and Wallace, 1979). In this context, GLU supplementation also enriched the relative abundance of *Papillibacter* spp., a bacterial group previously associated with enhanced N recycling in the rumen. *Papillibacter* spp. were more abundant in grazing Nellore cattle supplemented with higher levels of post-ruminal urea, suggesting a possible role in nitrogen turnover and urea hydrolysis (Souza et al., 2022).

Despite this apparent adequacy in ammonia N supply, no differences were observed in MPS or EMPS between treatments. This finding contrasts with previous *in vitro* studies that identified changes in MPS due to non-protein nitrogen sources in the diet (Zhu et al., 2022). Effective utilization of N by rumen microbes depends not only on ammonia N supply but also on the synchronization with fermentable energy sources (Poppi and McLennan, 1995; Ekinci and Broderick, 1997), which were likely constrained under the low-quality forage conditions of this study. The observed EMPS in both treatments (165 and 115 g MPS/kg RDOM) was below the 195 g MPS/kg RDOM value recommended by ARC (1984). This suggests that while N availability was sufficient in the rumen, other factors, such as limited energy availability due to high forage indigestibility as previously discussed, may have restricted microbial growth and consequently fermentation end products. Rumen bacteria need both ammonia and/or amino acids and energy substrates to maintain growth and MPS (Firkins et al., 2007). Our results are in line with the concept that microbial growth is limited by nitrogen and energy, an imbalance in these factors results in lower ruminal fermentation efficiency (Hackmann and Firkins, 2015).

PCA revealed that SMU appears to support microbial populations linked to transcriptional regulation, transport, and structural metabolism under conditions of low-quality forage. SMU microbial profile was characterized by higher relative abundances of the Firmicutes phylum and associated with bacterial groups *Ruminococcaceae UCG-002* and *Lachnospiraceae*. Firmicutes include several ruminal ammonia-producing bacteria (Bento et al., 2016), possibly stimulated by NPN availability. This phylum has been linked to enhanced feed and energy utilization efficiency, suggesting a microbiota profile more capable of utilizing fibrous carbohydrates (Turnbaugh et al., 2006). *Ruminococcaceae UCG-002* belongs to the well-recognized fibrolytic family Ruminococcaceae (Koike and Kobayashi, 2009). Similarly, members of the *Lachnospiraceae* play an important role in xylan degradation and are linked to cellulose-degrading microbes (Flint et al., 2008). These bacteria contribute to carbohydrate fermentation and the production of SCFA, which are essential energy sources for the host (Hernandez-Sanabria et al., 2010). This suggests that SMU supplementation, despite not enhancing nutrient digestibility in this study, promoted microbial communities adapted to structural carbohydrate degradation under the constraints of low-quality forage.

Supplementation with GLU as a source of RUP in grazing steers during the dry season, under conditions high indigestibility of the forage, modulated the rumen microbiota by increasing the abundance of key fibrolytic bacteria and enhancing metabolic predicted pathways related to amino acid utilization, glycan biosynthesis, and fermentative activity. These microbial shifts were accompanied by higher concentrations of BCVFA and improved fiber digestibility. However, these changes did not result in significant improvements in overall nutrient intake or microbial protein synthesis. While RUP supplementation demonstrated potential to enhance the functional capacity of the rumen microbiome, its benefits were limited by forage quality. Finally, future studies should confirm significant changes in predicted metabolic pathways through targeted metabolomics and gene expression analysis, while also integrating ruminal microbial and animal performance measurements to thoroughly evaluate the biological and production implications of these findings.

## Data Availability

The sequences of bacterial diversity have been deposited in the NCBI Sequence Read Archive (SRA) under accession number PRJNA1320702. The datasets generated for this study can be found in the public repository of UNESP, Brazil: http://hdl.handle.net/11449/204620.
